# Photopatterning of PDMS Films: Challenging the Reaction between Benzophenone and Silicone Functional Groups

**DOI:** 10.3390/ma14082027

**Published:** 2021-04-17

**Authors:** Arthur Stricher, Renaud G. Rinaldi, Laurent Chazeau, François Ganachaud

**Affiliations:** 1 Ingénierie des Matériaux Polymères, CNRS UMR 5223, Insa-Lyon, Univ Lyon, F-69621 Villeurbanne, France; arthur.stricher@gmail.com; 2 MATEIS, CNRS UMR 5521, INSA-Lyon, Univ Lyon, F-69621 Villeurbanne, France; renaud.rinaldi@insa-lyon.fr (R.G.R.); laurent.chazeau@insa-lyon.fr (L.C.)

**Keywords:** photoirradiation, hydrosilylation, platinum catalyst, inhibition, microfluidics

## Abstract

Direct photopatterning of PDMS (Polydimethylsiloxane) through benzophenone photo-inhibition has received great interest in recent years. Indeed, the simplicity and versatility of this technique allows for easy processing of micro-canals, or local control of PDMS mechanical properties. Surprisingly, however, the chemical reactions between silicone hydride and/or silicone vinyl groups and benzophenone have only been assessed through qualitative methods (e.g., Attenuated total reflection fourier transform infrared). In this communication, the previously proposed reaction pathways are challenged, using nuclear magnetic resonance (NMR) spectroscopy and size exclusion chromatography (SEC) monitoring. A different mechanism depicting the role of benzophenone irradiation on the polyaddition reaction of silicone formulations is proposed, and a simplified procedure involving aromatic solvent is finally disclosed.

## 1. Introduction

Benzophenone, also known as diphenyl ketone, is widely used in photochemistry (see e.g., [1]). It forms radicals upon ultraviolet (UV) irradiation below 365 nm, via the two symmetric and UV absorbing aromatic groups linked by the ketone group [2]. Its photoreaction with a large variety of molecules, such as alcohols [3,4,5], ketones [5] and aromatic solvents [4], has been studied extensively. In the field of silicone, it has been extensively used to promote radical grafting or polymerization of various hydrophilic monomers onto PDMS (Polydimethylsiloxane) [6], including acrylic acid [7] or N-isopropyl acrylamide [8], and more recently via controlled radical polymerization [9,10].

Bhagat et al. [11] reported an original use of benzophenone photoactivation to craft photopatternable silicone elastomeric films 15 years ago. Upon UV irradiation, benzophenone is believed to consume the reactive groups borne by the silicone chains, preventing crosslinking reactions promoted by heat curing. Using a mask, the sole irradiated part remains liquid during subsequent curing, and can then be removed from the silicone elastomer film by solvent washing to develop the targeted pattern. This technology has been further applied by the same group [12], and further reproduced worldwide [13,14,15,16,17,18] (see also a recent review on patterning of silicone surfaces [19]). According to the applications targeted by different teams, especially in biology-related works, it appears important to answer the following questions: 1. Is benzophenone definitively grafted to the siloxane chains? 2. Does benzophenone produce protruding and/or free polymer chains in the material?

**Figure 1:**
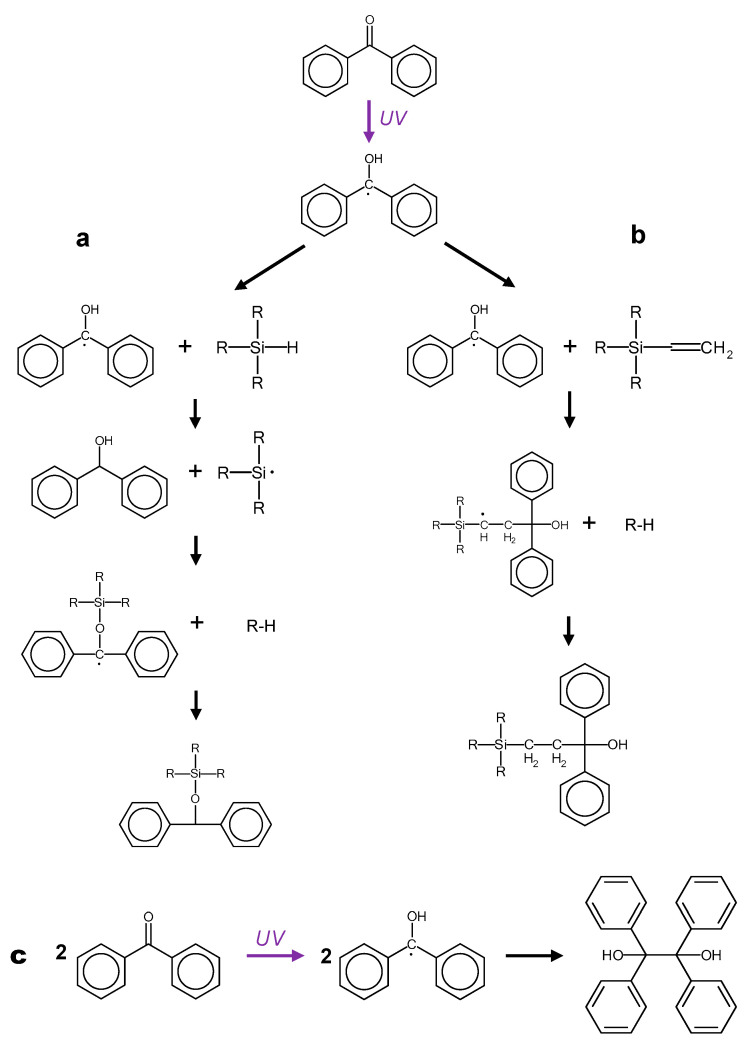
Proposed reactions occurring between UV-excited benzophenone and (**a**) Si-H and (**b**) Si-Vinyl functions [11]. (**c**) Acknowledged photoreaction of benzophenone on itself, forming benzopinacol [4].

The commonly accepted reaction mechanisms [11] involved in the photopatterning process are: (i) hydrogen abstraction of Si-H moieties by benzophenone radicals formed upon UV irradiation, described in Figure 1a; (ii) attack of the carbon double bond in vinyl moieties by the benzophenone radical, leading to the formation of short complexes, as depicted in Figure 1b. The monitoring of these reactions was performed exclusively and qualitatively by attenuated total reflection fourier transform infrared (ATR-FTIR) before and after irradiation, showing a small decrease of the C = O, C = C, and Si-H peaks. The C = O band decrease can arguably be accounted to the benzophenone radical formation, but benzophenone is also known to photo-dimerize, forming benzopinacol [4] (according to the scheme depicted in Figure 1c), which would generate equivalent features on the FTIR trace. One could also dispute that the observed C = C band decrease is small and might not be significant; whereas, the Si-H band’s small yet significant diminution, could be explained by other phenomena, the Si-H moieties being known for their instability and potential hydrolysis [20].

Here, a bi-component silicone formulation (similar to Dow Corning’s Sylgard 184, see experimental part) was used to first replicate the protocol developed by Bhagat et al. [10]; i.e., the photopatterning of a PDMS substrate containing benzophenone on a glass wafer. Once the experimental parameters of the UV irradiation (dose, irradiance) and curing (time, temperature) steps were identified, the chemistry of irradiated pristine silicone (benzophenone-free) was compared with the benzophenone-filled one to try to evidence the aforementioned reactions. The same experiment was then performed using simple model silicone molecules, containing either hydride or vinyl functions. Aliquots before and after irradiation were analyzed by several techniques, namely ^1^H, ^13^C NMR spectroscopy, and SEC (size exclusion chromatography) with toluene as an eluting solvent. A new mechanism is finally proposed on the basis of observed results.

## 2. Materials and Methods

### 2.1. Materials

Silicone formulation LSR Silbione 4360AB was kindly provided by Bluestar Silicone (Saint Fons, France) and used as received. Xylenes (ACS reagent ≥ 98.5%, mixture of isomers), 2-propanol (ACS reagent ≥99.5%), deuterated chloroform (>99.8% atom D), 1,1,3,3-tetramethylcyclotetrasiloxane (≥99.5%, labelled M_2_^H^), 1,3-divinyltetramethyldisiloxane (≥99.5%, labelled M_2_^Vi^) and benzophenone (ReagentPlus^®^ 99%) were purchased from Sigma-Aldrich (Saint Quentin Fallavier, France) and used as received.

### 2.2. Methods

Quantitative ^1^H NMR spectroscopy was performed on a BRUKER Avance III 400Hz spectrometer (Bruker Gmbh, Mannheim, Germany), equipped with a BBFO+ 5 mm probe for ^1^H analysis. Reactive moieties were normalized to the Si-CH_3_ peak, taken as the reference. Samples were dissolved in deuterated chloroform, at a concentration of 30 mg in around 0.6 mL of solvent. A total of 256 scans were recorded with a relaxation time of 2 s between each scan. Proton NMR spectra simulations were performed using the software Mestrenova 10.0.2 and associated Mnova NME plugin (MestreLab Research, Santiago de Compostela, Spain).

Molar mass measurements were carried out via size exclusion chromatography with toluene as an eluent. Samples were dissolved in HPLC grade toluene from Sigma Aldrich (Saint Quentin Fallavier, France) at a concentration of 3 mg/mL and then filtered on a 0.45 µm pore size. Signals were recorded with a Malvern Viscotek (Malvern Panalytical, Malvern, UK) apparatus equipped with 3 Shodex columns (Shodex China Co.,Ltd., Shanghai, China), and eluted at a 1 mL/min flow. Molar masses were evaluated using a conventional calibration with polystyrene standards, with signals from a Viscotek VE 3580 (Malvern Panalytical, Malvern, UK) refractive index detector.

### 2.3. Photopatterning of Silicone Formulation

The PDMS mixture was first prepared by mixing the same weight of parts A (containing silica, vinyl silicone oil and crosslinking catalyst) and B (containing silica, vinyl silicone oil, hydroxyl silicone oil as crosslinker, and cyclohexen-1-ol as a thermal inhibitor) plus 5 phr of benzophenone dissolved in xylene at a weight ratio of 3:5 in a Flackteck Inc speedmixer (Landrum, SC, USA) at the speed of 2750 rpm for 4 min. 3 × 3 cm glass wafers were cleaned using acetone, methanol, and DI water for 2 min followed by 5 min in sulfuric peroxide exposition, and finally dried with N_2_. Wafers where then dip-coated with the mixture, the bottom part wiped and xylenes were evaporated using a vacuum oven at room temperature, at a pressure below 10 mbar, for 30 min. Samples were subjected to UV irradiation thanks to an UVP CL-2000 crosslinker (Analytic Jena, Jena, Germany) equipped with five 245 nm wavelength UVc bulbs (Analytic Jena, Jena, Germany). The irradiation dose was measured by placing an EIT UV PowerPuck EIT II, (Budapest, Hungary) under the lit crosslinker for 5 min prior to irradiation, after a 5 min preheating of the lamps. The irradiation dose was set to be 8 J/cm^2^, at an average power of 2.8 mW/cm^2^, which coincides with the range explored in anterior published works [11,12]. A mask was used to cover half of the sample from irradiation, and crosslinking was then carried out using a 120 °C hot plate for 12 min. The (irradiated) uncured PDMS was finally removed by dipping the wafer in toluene for 10 s, then rinsed with isopropanol and dried with N_2_. This protocol was applied on pristine (reference) and benzophenone-filled silicone formulations. Samples to be analyzed in SEC and NMR were taken before and after irradiation, prior to the thermal curing step (un-crosslinked formulations).

### 2.4. Irradiation of Model Molecules

Benzophenone was mixed with M_2_^H^ or M_2_^Vi^ at a stoichiometry of 1 molecule of benzophenone per reactive (either vinyl or hydride) group. The blends were subjected to the same irradiation (dose and irradiance) as the formulated samples, and kept stirred continuously during the process to guarantee a good mixing. The depth of the solution was set to be smaller than 2 mm, to ensure a proper irradiation throughout the films’ thickness. Samples were taken before and after irradiation.

## 3. Results

For the sake of clarity, the following nomenclature is used to present and discuss the results: reference samples will be named *R*, and samples containing benzophenone *Z*, with the suffixes 0 and 1 for the un-irradiated and irradiated samples respectively. Therefore, Z1 stands for an irradiated sample containing benzophenone. Again, all characterizations were performed on uncured, i.e., un-crosslinked silicones.

### 3.1. Photopatterning Reproduction

**Figure 2:**
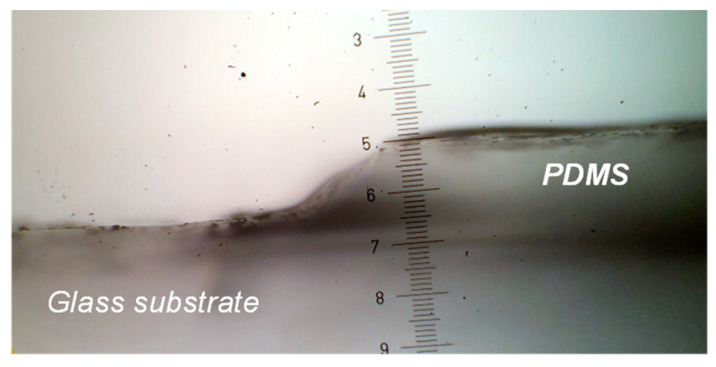
Topological photopatterning of silicon membranes, via irradiation and solvent wash of un-crosslinked zones (left side). The superimposed ruler gives an idea of the dimensions of the gradual transition from the obtained PDMS film to the naked glass substrate after washing, typically 2 cm wide.

As a preliminary experiment, we adapted the protocol developed by Bhagat et al. [11] to manufacture photopatternable PDMS thin films from a commercial silicone formulation crosslinking by polyaddition, similar to the Sylgard 184 previously used (see experimental part). Providing the proper irradiation and curing parameters, only the half part of irradiated, i.e., un-crosslinked, silicone coated on a glass wafer could be washed away. A picture of the transition zone between the irradiated and masked parts is presented in Figure 2. A gradual transition zone is clearly observed and has been fully explained in other studies [17]. When the reference sample, containing no benzophenone, was prepared under the same conditions (half-masked, irradiated and cured), the crosslinking occurred in the entire film, evidencing if needed the active role of the benzophenone in the process (not shown).

### 3.2. Analyses on LSR Formulations

**Figure 3:**
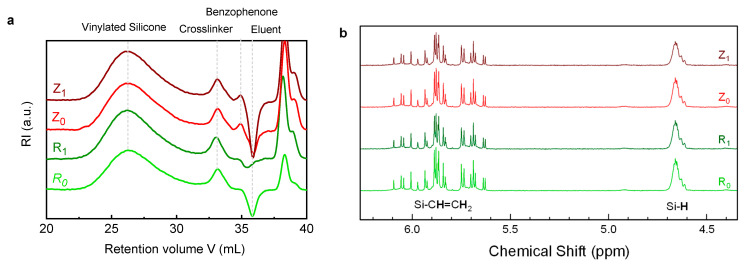
(**a**) Offset SEC traces and peak attribution; (**b**) Offset proton NMR focused on the reactive moieties zone.

The study of the reaction preventing the crosslinking, presumably occurring between the benzophenone and the PDMS polymer, was deepened by performing SEC and NMR measurements on aliquots retrieved at different steps during the process. As seen in Figure 3 and Table 1, SEC traces of pristine silicone display two peaks, at low retention volume (high molar masses) and high retention volume (low molar masses) [21]. The high molar masses peak accounts for vinylated silicone, which corresponds to the matrix of Liquid Silicone Rubber (LSR) and has a weight average molar mass of 137,000 g.mol^−1^. The smaller one is attributed to the crosslinker, made of smaller molecules of poly(dimethylsiloxane-co-hydrogenomethylsiloxane) with *M_w_* around 1600 g.mol^−1^ (Table 1). Quantitative proton NMR spectra of pristine silicones are displayed in Figure 3b, focusing on the hydride and vinyl moieties, and the corresponding values in Table 1. A quick estimation of the number of units per chain is 8 vinyl groups per long chain, and about 10 Si-H per small chain.

When the irradiation takes place in the presence of benzophenone (i.e., Z1), both crosslinker and vinylated silicone chains are expected to increase in mass through its grafting onto the polymer chains, according to the proposed mechanism. The molar mass of benzophenone is 182 g.mol^−1^, so we expect that at full conversion, the grafted benzophenone units would increase the molar mass of both macromolecules by about 1500 g/mol. This shift is not expected to be visible for the vinylated chains, according to the precision of the SEC device (estimated at 5%) and the large molar mass of the chains. Indeed, the molar mass of the vinylated chains varies by less than 4% amongst the samples, showing no significant modification considering the uncertainty of the measurement (Table 1).

On the other hand, such variation should be visible on the low molar mass Si-H functionalized chains. The crosslinker’s *M_w_* indeed slightly increases with irradiation; however, this is regardless of the presence of benzophenone. Since the increase in molar mass of the samples containing benzophenone (*Z* series) is less than 110 g.mol^−1^, below the molar mass of benzophenone itself, it means that less than one molecule of benzophenone on average would be grafted to each crosslinker chain. Ultimately, such low functionalization would not allow preventing further silicone curing under heat.

**Figure 4:**
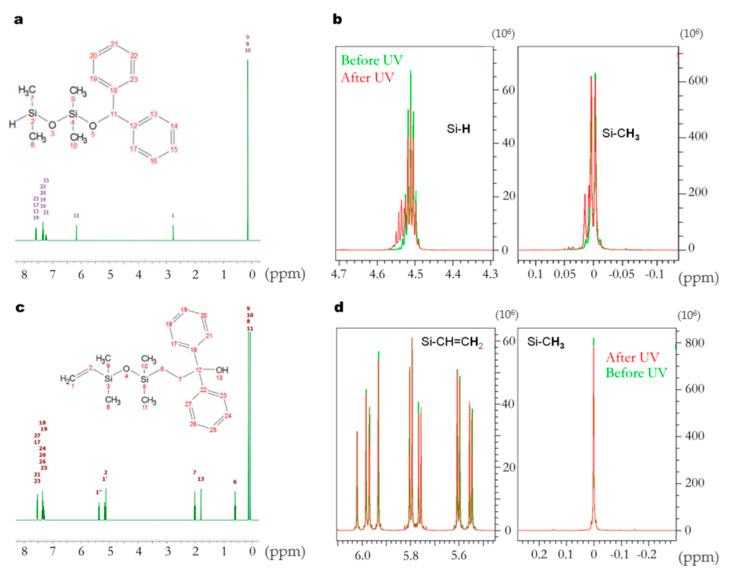
(**a**) Simulated proton NMR spectra of M_2_^H^ reacting with benzophenone. (**b**) Proton NMR spectra prior (in green) and after (in red) irradiation of M_2_^H^ + benzophenone mixture. (**c**) Simulated proton NMR spectra of M_2_^V^ reacting with benzophenone. (**d**) Proton NMR spectra prior (in green) and after (in red) irradiation of M_2_^Vi^ reacting with benzophenone. Note that the peaks are normalized by superimposing peaks of the Si-CH_3_.

To confirm such a hypothesis, quantitative proton NMR spectra of all formulated silicones were carried out. Figure 4a,c show a simulation carried out on model oligomers, namely 1,1,3,3-tetramethylcyclotetrasiloxane (M_2_^H^) and 1,3-divinyl-tetramethyldisiloxane (M_2_^Vi^), respectively. Here, only one end-chain is assumed to have reacted. If a photoreaction occurs between benzophenone and siloxane radicals, new peaks should appear on the spectra of the irradiated samples, such as Si(Me)_2_-CH_2_-CH_2_-C-OH(φ)_2_ (expected at 0.8 to 2 ppm, Figure 4a) when reacting with vinyl moieties, or Si-O-CH-(φ)_2_ (at around 6 ppm, Figure 4c) when reacting with hydride moieties. None of these peaks were observed on the NMR spectra of irradiated formulations. Attention was then specifically focused on the vinyl and SiH regions of the LSR products (Figure 3b). Again, no noticeable changes between irradiated (*R*1 and *Z*1) and non-irradiated samples (*R*0 and *Z*0) were observed. Additionally, the integration values of the peaks of the vinyl and the hydride groups, quoted in Table 1, show almost no changes whether benzophenone is present or not, or whether irradiation occurred or not. One might argue that there is a small diminution of the number of Si-H in the *Z*1 sample, yet less than 4%, which is below the commonly accepted uncertainty of NMR dosage, typically 3% (vide infra).

### 3.3. Study on Model Molecules

From these preliminary studies, we concluded that benzophenone reacts neither on vinyl nor Si-H groups. Thus, to better understand the crosslinking inhibition, we have irradiated mixtures of benzophenone and previously simulated model oligomers, M_2_^H^ and M_2_^Vi^, to simplify the plot. The results are displayed in Table 2 and Figure 4d,e.

For M_2_^Vi^, the peaks in the Si-vinyl and Si-CH_3_ region fully overlap before and after irradiation, so there are no reactions occurring on either of these functions. For the sample with M_2_^H^, there is a drop of 10% in the amount of hydride after irradiation. On the NMR spectrum of the irradiated samples, the Si-H massif around 4.5 ppm is enlarging towards larger chemical shift, whereas in the Si-CH_3_ region, two new peaks appear in the massif at 0.005 and 0.015 ppm. These shifts are typical of a change of environment of these groups because of partial Si-H transformation. Hydrolysis can be one reaction taking place, hydride moieties being known for their relative instability [22]. This is actually why these functional groups are commonly and voluntarily introduced in excess in silicone elastomer formulations—so that a sufficient amount remains despite the crosslinker’s aging [21]. Another reaction could be a random radical activation of the highly reactive Si-H moieties by UV. Nevertheless, this second reaction remains unlikely since precise conditions, in particular specific platinum catalysts, are required that are not fulfilled here [23].

## 4. Discussion

All in all, the results tend to show that benzophenone reacts neither with vinyl nor hydride groups from the silicone polymers, conversely to what is assumed in Figure 1. Furthermore, the main chemical functions remain globally intact after irradiation, calling for a new explanation for the crosslinking inhibition.

Apart from silicone chains, basic LSR silicone formulations contain the following components [21,24]: fillers (e.g., fumed silica), a crosslinking catalyst (usually a platinum complex), and possibly a thermal inhibitor (often ethynyl cyclohexanol). There are no reasons that the filler be affected by UV irradiation. Moreover, should the thermal inhibitor be degraded by the irradiation, it would favor crosslinking rather than preventing it. Therefore, the most plausible explanation to the observed absence of crosslinking in irradiated silicone is that the catalyst is sensitive to UV and deteriorates when exposed to these energetic rays.

The photo-instability of Karstedt’s catalyst, the most widespread catalyst used for hydrosilylation, has been previously reported by Lappert et al. [25], and similar platinum or metallic complexes are known to be subject to decomplexation and colloid formation when subjected to energetic rays [26,27]. This scenario also explains why the originators of PDMS curing inhibition by UV irradiation [11] observed some crosslinking when overbaking their irradiated photo PDMS films. Indeed, assuming the existence of stable molecular arrangements through reactions between benzophenone and functional groups of the silicone, overbaking would not have been an issue.

This scenario is supported by our recent results, where we patterned silicone films in the presence of aromatic solvent (typically Xylenes) [28]. In this article, we demonstrated that the crosslinking catalyst is degraded when irradiated in the presence of an UV-absorbing solvent, even in amounts as small as one percent in mass. We showed that such degradation into inert colloidal platinum slows down the crosslinking kinetics, thus depressing the final mechanical properties of irradiated film. Hydrosilylation can be accelerated again by an increase of temperature or an augmented duration of the post-curing treatment of the irradiated silicone. Similarly, the benzophenone is an aromatic molecule and as such, it can absorb light to likely provoke the degradation of the crosslinking catalyst. The same mechanism, therefore, can explain the inhibition of crosslinking observed here, with the major drawback being that this molecule is not easily extracted from the final silicone elastomer, compared to volatile xylenes, and finally generates colored patterned films.

## 5. Conclusions

This study challenged the reported explanation for the observed crosslinking inhibition of PDMS when irradiated in the presence of benzophenone, prior to the crosslinking reaction. The absence of variation of molar masses and number of reactive groups with or without UV irradiation indicate that no chemical reactions occur between benzophenone radicals and PDMS reactive groups, in contrast to accepted theories. Another phenomenon explains the observed inhibition of crosslinking, namely, the photo-degradation of the crosslinking catalyst into inert colloidal platinum when exposed to UV rays. The reduction of active catalyst content depends on the time of irradiation. Nevertheless, even at very depressed platinum content, the crosslinking rate is slowed down but not totally inhibited and PDMS can finally be crosslinked by applying a longer curing time. This scenario tends to further demonstrate why the curing time, rather than the irradiation dose or the stoichiometry of benzophenone to reactive radicals, is such a crucial point in the process first presented by other research teams. We are currently trying to adapt this process to the photopatterning of RTV formulations [29,30].

## Figures and Tables

**Table 1:** SEC and NMR analyses on LSR formulation without (R) and with (Z) benzophenone that has been irradiated (1) or not (0).

Samples	SEC				NMR		
	Crosslinker + Oils		Vinylated Chains		n_SiVi_^a^	n_SiH_^a^	n_SiVi_/n_SiH_^a^
	M_w_ (g·mol^−1^)	Đ	M_w_ (g·mol^−1^)	Đ			
R0	1570	1.4	134,000	2.3	4.26	7.72	1.81
R1	1900	1.3	139,000	2.3	4.24	7.63	1.80
Z0	1640	1.4	138,000	2.3	4.27	7.72	1.81
Z1	1750	1.4	136,000	2.3	4.23	7.40	1.75

^a^ Number of functional units per 1000 dimethylsiloxane units, determined by ^1^H NMR, via the relative integration with normalized spectra according to the SiCH_3_ unit.

**Table 2:** NMR analyses for model molecules.

	UV	NMR			
		n_SiMe_^a^	n_reactive moieties_^a^	r_calculated_^b^	r_theoretical_^b^
M_2_^H^ + Benzophenone	0	4	1.85	2.16	2
	1	4	1.67	2.39	2
M_2_^Vi^ + Benzophenone	0	4	1.87	2.14	2
	1	4	1.88	2.13	2

^a^ Number of functional units per 1000 dimethylsiloxane units, determined by ^1^H NMR, via the relative integration with normalized spectra according to the SiCH_3_ unit; ^b^ ratio of n_SiMe_/n_reactive moieties._

## Data Availability

Data is contained within the article.
